# Social Attention Through a New Lens: Autistic and ADHD Traits and Eye Occlusion Affect Gaze During Conversation Watching

**DOI:** 10.1177/17470218251390498

**Published:** 2025-10-16

**Authors:** Jessica Dawson, Astrid P. Martinez-Cedillo, Leilani Forby, Bradley Karstadt, Alan Kingstone, Tom Foulsham

**Affiliations:** 1 Department of Psychology, University of Essex, Colchester, UK; 2 Department of Psychology, University of York, UK; 3 Department of Psychology, University of British Columbia, Vancouver, BC, Canada

**Keywords:** Social attention, eye movements, autism, attention deficit hyperactivity disorder

## Abstract

When people watch a pre-recorded conversation, they tend to follow the speaker and spend most of the time looking at the faces and eyes of those talking. To test whether these responses reflect realistic social attention, we eye-tracked participants in two experiments where the depicted speakers sometimes wore sunglasses which removed fine-grained information from the eye region. We also examined clinically relevant traits which have been shown to have an effect on social attention, by including individuals with high- and low- levels of traits associated with autism (Experiment 1) and Attention Deficit Hyperactivity Disorder (ADHD; Experiment 2). Those with high levels of autistic traits were less likely to look at key facial features. ADHD symptoms did not have the same effect. When there was a change in speaker, sunglasses disrupted attention such that there were fewer and later looks to the new speaker. Being able to read gaze cues, therefore, facilitates attention in conversation, and subtle differences in this behaviour may be associated with clinically relevant traits.

## Introduction

Following a conversation is a difficult, multisensory task. Yet humans typically engage successfully in conversation, forming a key part of the daily interactions that are important for our social and professional lives. Understanding these interactions, and taking part in them, involves responding to cues that can, for example, tell us whose turn it is to speak next.

In the present research, we test whether responses in these social interactions can be captured in a task where participants watch pre-recorded videos. In particular, if responses to video-recorded conversation are truly “social” then we would expect them to be sensitive to eye gaze from the people in the video. In real interactions, we readily use the gaze of others to regulate our turns when speaking (Ho et al., 2015; Kendon, 1967). However, gaze between individuals does not function in the same way on video as in real life (Laidlaw et al., 2012). Here, we test the sensitivity to gaze cues by occluding the eyes with sunglasses in one condition.

As a second test of the social relevance of video-recorded conversations, we also investigate differences between individuals with different levels of clinically relevant traits. We begin by reviewing the role of the eyes in social attention.

### Signalling with the Eyes

Human beings tend to focus on the faces and eyes of others (Emery, 2000). The eyes of another provide us with information about intentions, emotions, and beliefs (Birmingham et al., 2007) and are habitually used to direct attention (“gaze cueing”). Recent research has confirmed that the eyes are crucial to signalling with a partner during live interactions and are involved with understanding transitions in turn-taking during conversation (Hessels, 2020; Ho et al., 2015; Macdonald & Tatler, 2018).

Ho et al. (2015) explored gaze in dyadic, face-to-face conversations. Their findings validated previous evidence suggesting that gaze functions as a signalling mechanism for who will speak next (Kendon, 1967). For example, speakers ended their turn with direct gaze at the listener. When the listener takes over, they begin speaking with averted gaze. This study, therefore, highlights the use of eye movements to signal when we would like to be listened to and when we would like a response. Indeed, it has been suggested that the morphology of the human eye has evolved to assist with such communication (Tomasello et al., 2007). The human eye has a white sclera with a coloured iris. The resulting contrast enables others to easily gauge looking direction and infer what we are attending to.

In natural conversation, it is rare that the eyes are observed alone without accompanying head movements and gestures. One way to isolate the role of the eyes in social attention is by occluding them with sunglasses. Occluding the eyes has been investigated in studies examining face recognition (Hockley et al., 1999) and expression identification (Roberson et al., 2012). In these studies, occluding the eyes with sunglasses impeded typical processing. Occluding the eyes will also remove gaze cues, and in a previous study, sunglasses led to poorer coordination in human–human and human–robot reaching tasks (Boucher et al., 2012). However, while it is clear that gaze is important when partners are working together with shared objects (e.g. to resolve ambiguity in speech; Hanna & Tanenhaus, 2004), it is less clear how much it is required when conversing without a shared reference.

Social interactions and eye contact may be disrupted in those with autism and Attention Deficit Hyperactivity Disorder (ADHD). Below, we review what is known about social attention in these groups.

### Social Attention in Autism

Participants with autism, as well as non-diagnosed individuals with high levels of autistic traits, show characteristic differences in eye contact in face-to-face situations (Chita-Tegmark, 2016). These unusual patterns have clinical relevance as such individuals may interact in less efficient or inappropriate ways and find it harder to read social cues from their interlocutors. If watching videos of a conversation can be considered a social task, we should therefore be able to identify effects of autistic traits – on responses to faces and eyes in the videos and on the effect of eye occlusion.

Alongside reduced social interaction, atypical eye contact is among the diagnostic criteria for autism (American Psychiatric Association [APA], 2013; Baron-Cohen, 1995; Jones et al., 2016). In the lab, autistic individuals tend to not look at people in images and movies to the same degree as typical participants (Dalton et al., 2005; Klin et al., 2002). Indeed, a considerable amount of research has investigated the extent to which autistic people show different eye movements in social contexts (Chita-Tegmark, 2016; Seernani et al., 2021.; Tye et al., 2013). Although there is consensus that autistic participants show fewer fixations on the eyes (Chita-Tegmark, 2016), there are mixed findings on where they choose to attend instead (e.g. other body parts or an object). For instance, Scheerer et al. (2021) examined whether autistic children have a stronger prioritisation towards trains over faces. Participants indicated whether a butterfly target was present or absent within an array of distracting stimuli (trains and faces). The results showed no difference in the degree to which autistic children were distracted by the two types of stimuli. Indeed, although the consensus from meta-analyses is that individuals with autism are less likely to fixate faces and eyes (Chita-Tegmark, 2016; Frazier et al., 2012), there are a number of published studies failing to find such a difference (Falck-Ytter et al., 2015; Kwon et al., 2019).

In the present study, we use a video of a real conversation. It has been suggested that it is in more complex settings that differences between autistic and non-autistic participants will emerge (Freeth & Bugembe, 2019; Hanley et al., 2013). Freeth et al. (2013) measured the time spent viewing a confederate when in a face-to-face interaction or on a pre-recorded video and correlated this with traits of autism in the general population. Their findings demonstrated that those with higher levels of autistic traits looked less to people when watching videos. However, interestingly, there was no difference in the live situation. Freeth et al. (2013) suggest that this may be because those with autistic traits were less interested in faces in the video condition, but that in real interaction, gaze was driven by the norms of the situation.

Measures of autistic traits in the normal population are used in the early stages of diagnosis (Allison et al., 2012), and these measures have also been shown to be associated with differences in social attention (Freeth et al., 2013; Vabalas & Freeth, 2016). However, we have also recently reported no difference between groups with high versus low traits of autism in a task involving watching pre-recorded conversations (Forby et al., 2023). Both groups showed similar gaze behaviour and also made the same judgements about the people depicted. In the present study, we reinvestigate any potential differences in groups from the normal population, and we ask whether such differences are affected by occluding the eyes. If we can capture truly social responses in a video-watching task, then we should be able to detect effects of autistic traits.

One consideration in the study of autism is the high estimated prevalence rate of comorbid psychiatric disorders. Some of the cognitive differences and symptoms of ADHD overlap with those seen in autism (Jang et al., 2013; Seernani et al., 2021). Seernani et al. (2021) examined eye movements in participants with ADHD, autism, autism + ADHD, and a Control group. They found that the autism group had better performance in a visual search task. The autism + ADHD participants were slower, inefficient, and had longer fixations. Performance in the ADHD individuals was normal. The authors suggested that the comorbid group should be seen as a separate group with its own symptoms, rather than as an addition of the autism and ADHD groups. In this study, we attempted to separate autism and ADHD traits, within a community sample.

### Social Attention in ADHD

ADHD is a neurodevelopmental disorder affecting 3.5% of school-aged children and showing a reduced prevalence in adults (APA, 2013; Faraone, 2000). The DSM-5-TR classifies three presentations on the basis of differences in symptoms: inattentive, hyperactive/impulsive, and combined (APA, 2013).

Atypical behaviours in ADHD have been uncovered with eye-movement analysis (Panagiotidi et al., 2017; Seernani et al., 2021; Van der Stigchel et al., 2007). For instance, Panagiotidi et al. (2017) demonstrated that people with high traits of ADHD have a greater number of microsaccades while maintaining fixation. Van der Stigchel et al. (2007) used an attentional capture task with children with ADHD, their unaffected siblings and a Control group. They reported that ADHD children had slower saccades than the Control group despite similar accuracy. Saccade latency and the proportion of intrusive saccades were related to the continuous dimensions of ADHD symptoms. A recent meta-analysis confirmed that participants diagnosed with ADHD show disturbances in eye movement control, particularly related to saccade inhibition (Maron et al., 2021). These studies suggest that atypical eye movement behaviour might be more evident in those with greater severity of ADHD symptoms.

There is relatively little research investigating social attention in ADHD. That which exists has used pictorial stimuli depicting social situations to understand emotion identification (Tye, et al., 2013) or gaze cueing (Marotta et al., 2014). Marotta et al. (2014) used three different conditions (eye gaze, arrows and peripheral onset cues). Participants were asked to detect a target which was either congruent or incongruent with a cue. They did not find differences between groups in the arrow and the peripheral onset cues conditions. However, when a gaze cue was presented, typically developing participants performed quicker in the congruent condition relative to the incongruent. This effect was absent for participants with ADHD. This suggests that gaze following is disrupted in ADHD. Serrano et al. (2018) also compared eye movement behaviour in ADHD and control groups but in an emotion identification task. The authors used images showing different facial expressions. They found that participants with ADHD spent less time looking at the face, and specifically the eyes and mouth. In addition, the ADHD group had slower reaction times than the control group when identifying the emotions.

These findings suggest that the general attentional impairments found in ADHD may also be seen for social stimuli, which could be significant given the comorbidity with autism. However, to our knowledge, there is no prior work investigating the impact of individual differences in ADHD symptoms in complex dynamic situations such as the real conversations presented here.

### Present Study

In the present study, we investigate the effect of occluding the eyes with sunglasses when observing a pre-recorded conversation (in half the clips the conversants are wearing sunglasses, in the other half, they are not). We use a similar methodology to Dawson and Foulsham (2021), where participants watched video clips depicting individuals without sunglasses engaged in a discussion. These stimuli are complex and realistic, requiring participants to pay attention to audio–visual cues (e.g. speech and gesture), and they can mimic real situations where individuals might find it hard to pay attention. Of course, watching video clips does not provide a true social experience because the people depicted cannot interact with the observer. As a result, a number of studies have shown different looking patterns, and in particular, fewer fixations on people in real situations compared to video (Laidlaw et al., 2011; López et al., 2023). However, for the stimuli used here, there is evidence that the spatiotemporal gaze behaviour displayed when watching these clips is similar to those in a real conversation, despite not being real interactions which involve the eye-tracked participant (Dawson & Foulsham, 2021). Here, we seek to test whether observers are using the information from the eye region when watching such clips.

We aimed to understand how populations with high and low traits of autism (Experiment 1) and ADHD (Experiment 2) view these interactions, and how they might be affected by the presence or absence of eye gaze cues. It is important to note that we report eye-tracking data collected from a subclinical sample who display symptoms but do not meet the criteria for the disorder per se. Notwithstanding this caveat, this subclinical sample might facilitate the understanding of these conditions, since it is less likely that participants will have taken psychostimulants or been under other clinical interventions.

We have three main objectives. First, we explore the extent to which the looks to people and key facial features (eyes and mouths) are affected by sunglasses occluding the eyes. Previous research indicates that typical participants will look at people within a video and particularly at their eyes (Foulsham & Sanderson, 2013). If the purpose of looking at the eyes is to understand intentions or emotions, then we may expect a decrease when occluded with sunglasses, as there is no additional benefit to be gained by fixating this area. In contrast, if we see no difference when the eyes are occluded, it may be because looking at the eyes is habitual.

Second, we assess how and when a speaker is fixated. This permits a more detailed test of the eyes’ role as a signalling cue. In a typical population, the majority of fixations in video tend to be on the person currently speaking (Dawson & Foulsham, 2021; Foulsham et al., 2010). Changes in speaker are often associated with gaze cues that act to make turn-taking smooth within a real interaction (Ho et al., 2015). This leads to the hypothesis that occluding the eyes with sunglasses will lead to differences or delays in following the conversation. This will be investigated in terms of looking behaviour to those currently speaking and in a time-based analysis to assess the moment at which a speaker is fixated.

Third, we assess individual differences in these patterns by comparing findings in those with high and low traits of autism and ADHD. For autism, we test the hypothesis that looking to the eyes and face will be reduced in high-trait individuals. If this is the case, then we might also expect a reduced effect of sunglasses, since these individuals will be less reliant on cues from the eye region. One possibility is that autistic individuals avoid the eyes because they are aversive (Freeth & Bugembe, 2019; Kliemann et al., 2012). Sunglasses might therefore benefit high-trait individuals, making their behaviour more similar to low-trait observers. It has previously been reported that autistic people may treat actors wearing sunglasses differently (Cañigueral & Hamilton, 2019). Specifically, in that study typical observers preferred to watch videos where the eyes were visible and the actor could see, whereas autistic observers sometimes preferred videos with sunglasses. Occluding the eyes in some videoclips will allow us to isolate the “stimulus-driven” aspect of social attention (i.e. the system which is activated by the presence of eyes).

The predictions for those with ADHD symptoms are less clear, but since shifting between speakers at the correct time requires a high level of attentional control, we might expect this behaviour to be impacted. Gaze cueing may be disrupted in ADHD (Marotta et al., 2014), and if such cues are important then we should expect differences when the eyes are not occluded. Moreover, if looking to the eyes and face is a rather automatic response (Laidlaw et al., 2012), then those with high traits of ADHD may find it more difficult to inhibit this response (for example, when the eyes are less informative in the sunglasses condition).

Collectively, this work will allow us to determine if this novel paradigm, which involves manipulating eye occlusion of pre-recorded conversations, is sensitive enough to detect reliable differences in social attention between different groups of individuals.

## Experiment 1

Experiment 1 was designed to examine eye movements in participants with high and low traits of autism while watching pre-recorded conversations, with the additional manipulation of occluding the target’s eyes.

## Method

### Transparency and Openness

We report how the sample sizes were determined, all manipulations, and all measurements in this study. All data for the present experiments have been made publicly available on Open Science Framework and can be accessed at https://osf.io/c3jvk/. Experiment 2 was pre-registered.

### Participants and Autistic Traits Classification

We classified autistic traits using a questionnaire in a large sample, and then invited a subset of these for the eye-tracking study. As part of the pre-screening questionnaire, more than 2,500 Psychology students at the University of British Columbia completed the AQ-10 questionnaire (Allison et al., 2012) when beginning the semester. The AQ-10 includes 10 items from the full Autism Quotient (AQ), and is used by some practitioners to decide if someone should be referred for a diagnostic assessment. Each item is scored according to whether the participant agrees with that behaviour or experience, and Allison et al. (2012) report good internal reliability and comparable performance to the full AQ. Our eye-tracked participants were selected from this population if they had either low levels of autistic traits (LAQ), where they had a total score of less than two, or high levels of autistic traits (HAQ), where they had a score of six and above (which is a cutoff suggested by Allison et al., 2012, for referral for a full diagnostic assessment). We invited participants to the lab only if they met these criteria (a fact that was not shared with the participants), with those who responded scheduled for testing until our sample size was met.

In the HAQ group (*N* = 21), there were 15 females (ages 18–24, *M* = 19.86, *SD* = 1.85), and in the LAQ group (*N* = 20) there were 19 females (ages 20–23, *M* = 21.25, *SD* = 1.02). This sample size was determined in advance, using heuristics (Lakens, 2022), as a compromise between power considerations and feasibility. A power sensitivity analysis (Lakens, 2022) indicates that this sample size is sufficient for detecting small-to-moderate within-subjects effects (*dz* > 0.45), consistent with those observed previously (Foulsham & Sanderson, 2013). The same analysis suggests that a simple comparison with 40 participants is sensitive to between-group differences with *d* > 0.91. This sample size is similar to that from recent studies comparing low/high trait groups (Forby et al., 2023; Vabalas & Freeth, 2016). Our design has increased power because we selected extreme groups based on our pre-screening, meaning that any difference according to traits will be more easily detected (Preacher et al., 2005).

All of the subjects reported normal or corrected-to-normal vision, and none reported a formal diagnosis of autism or other neurodevelopmental disorders. Participants were granted with course credit for their participation and gave their written consent. We received ethical approval from the University of British Columbia.

### Apparatus

Eye position was recorded using the Eyelink 1000 (SR Research Ltd., Mississauga, Canada), a video-based eye tracker that samples pupil position at 1000 Hz. A nine-point calibration and validation procedure was repeated several times to ensure that all recordings had a mean spatial error better than 0.5°. Head movements were restricted using a chin rest, and sound was played through headphones. Participants sat 60 cm away from the monitor so that the stimuli subtended approximately 38° × 22° of visual angle. Saccades and fixations were defined according to Eyelink’s acceleration and velocity thresholds.

### Stimuli

The stimuli were video clips showing realistic conversations, and which were specifically recorded for this study. The video clips depicted six target individuals having a discussion while sitting around a table, with only three individuals (one side of the table) in view in each clip. The clips were derived from a longer recording with a static video camera, which is a permanent feature of the Observation Laboratory at the University of Essex. The targets were two groups of males and two groups of females, six people per group, who were all members of various sports teams at the University of Essex. Targets were given various questions, in a randomised order, which they were to discuss as a group (e.g. “what is your most embarrassing moment?”). These were questions or topics designed to enable natural conversing by all team members.

Each group was given sunglasses to wear for some of the interaction. Targets were given an equal number of questions to discuss with and without sunglasses and conditions were counterbalanced in terms of order and questions. When given the sunglasses, all six people wore them, and they were told that this was to examine how they behave when wearing sunglasses.

**Figure 1.**
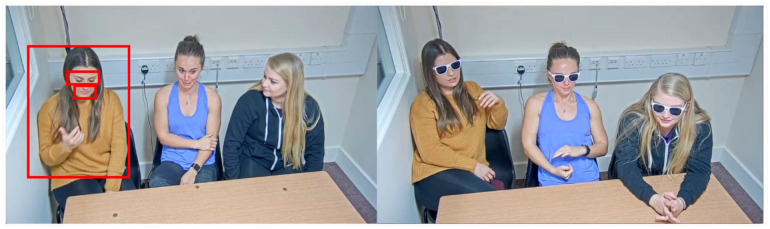
Example video frames from the control (left) and sunglasses (right) conditions, showing three of the target individuals. The target, eye and mouth ROIs are illustrated for one person only. *Note.* ROI = Region of interest.

Experimental clips were selected from each continuous recording and featured moments where all visible targets spoke at least once and the targets in view were the predominant speakers, with minimal involvement from the people on the other side of the table. Two 35 s clips were chosen from each group, one with sunglasses and one without (which we hereafter refer to as the control condition). The result was eight experimental clips (see Figure 1 for examples). Since both sets of clips were recorded from the same people, at the same time, in the same task and setting, they were well matched and included both male and female targets equally. We also selected clips for each condition which had similar content and distribution of speakers.

### Procedure

The eye-tracked participants read and completed consent forms and were asked to confirm that they had normal or corrected to normal vision before beginning the experiment. After the participant’s right eye had been successfully calibrated and validated, the experiment began. The eight experimental trials were presented in an interleaved, randomised order. Each trial started with a fixation dot displayed for 500 ms followed by the 35 s long video clip. After each clip, six questions were presented which asked about the people or events in the video clip. (e.g. “which person was wearing a green t-shirt?”), and participants responded by pressing “1,” “2,” or “3” on the keyboard to indicate one of the three targets. The questions were piloted for difficulty before beginning the study, and only used to ensure participants were paying attention to the clips.

### Data Analysis

Trials for each participant were checked for periods where eye-tracking data were missing, but no trials or participants were removed on this basis. We then extracted key statistics for each trial using EyeLink’s Data Viewer software (SR Research). Measures were averaged across trials, within each condition, and participant means were submitted to repeated measures analysis of variance (ANOVA). No deviations from the assumptions of ANOVA were noted.

Our results concentrate on fixations to important regions of the face and their timing in the conversation. Details of general viewing behaviour can be found in the accompanying Supplemental Information. In general, both groups made the same number of fixations and spent almost all the time fixating the people.

Fixations on targets were defined with a region of interest (ROI) around each target. Target ROIs subtended approximately 10° × 11.5° of visual angle but varied slightly with the size of the target (see Figure 1 for an example of all ROIs). Previous studies have found a tendency for typical observers to fixate the eyes in images and video (Birmingham et al., 2007; Dawson & Foulsham, 2021; Klin et al., 2002). For this reason, we investigated differences in looks to specific regions of the face. Rectangular ROIs were drawn around the eyes and mouth of each of the three targets. The positions throughout the recordings were adjusted by slowly playing the clip back with “mouse record” (an inbuilt function in Data Viewer), which allowed the tracking of these areas when targets moved. In all cases, we made ROIs as large as possible without overlapping the key features (Hessels et al., 2016). The average dimensions of the eye and mouth ROIs were 3.5° × 1.2° and 2.0° × 1.0°, respectively, and this did not differ between control and sunglasses conditions.

We report the proportion of fixations on a region as our main outcome measure. In this experiment, there was a strong correlation between the number/proportion of fixations on a region in a given trial and the total amount of time spent on that region (which can be called total dwell time). In the Supplemental Information, we repeat our analyses using the proportion of dwell time, and this leads to the same conclusions reported here.

## Results

### Fixations to Targets’ Eyes and Mouth

Table 1 shows the average percentage of fixations on the eyes, mouth, and elsewhere on the target (i.e., the body and other regions of the face).

**Table 1.** The Mean Percentage of Fixations to Targets’ Eyes and Mouth, Split by Group (Low and High Levels of Autistic Traits) and Condition.

Group	Statistic	Mean % fixations to targets					
		Control condition			Sunglasses condition		
		Eyes	Mouth	Elsewhere	Eyes	Mouth	Elsewhere
HAQ	M	25.27	14.29	60.44	41.47	15.94	42.59
	SD	15.77	13.42	13.05	21.77	11.72	19.48
LAQ	M	32.24	17.38	50.37	45.59	23.17	31.24
	SD	15.25	14.08	8.08	20.00	15.46	15.58

*Note.* Fixations outside the main target ROIs are not included here, and thus percentages within a condition sum to 100%. ROI = Region of interest; HAQ = High levels of autistic traits; LAQ = Low levels of autistic traits.

Participants’ average percentages were entered into an ANOVA with the within-subject factors of condition and area (mouth and eyes) and the between-subjects factor of group. There was an effect of area (*F*(1, 39) = 17.516, *p* < .001, η^2^ = .096), indicating that participants fixated more to the eyes compared to the mouth. There was an effect of group (*F*(1, 39) = 6.266, *p* = .017, η^2^ = .138), indicating that the LAQ group made more fixations to both areas in comparison to the HAQ group. This confirms the hypothesis that high levels of autistic traits will lead to reduced attention to the face. There was also an effect of condition, (*F*(1, 39) = 122.389, *p* < .001, η^2^ = .758). Interestingly, this was qualified by an interaction between condition and area (*F*(1, 39) = 29.804, *p* < .001, η^2^ = .433), indicating that the bias to look at the eyes rather than the mouth was more pronounced in the sunglasses condition compared to the control condition. There was no interaction between area and group (*F*(1, 39) = 0.002, *p* = .965, η^2^ < .001) or between condition and group (*F*(1, 39) = 0.146, *p* = .704, η^2^ = .004). The Three-Way Area × Condition × Group interaction was also not reliable (*F*(1, 39) = 2.983, *p* = .092, η^2^ = .071). These non-significant interactions indicate that the presence of sunglasses did not change the effect of autistic traits.

### Fixations to Speakers

Fixating the person who is currently speaking appears to be a common pattern of behaviour, and so we can also ask whether this is disrupted in high-trait individuals or by the masking of gaze cues with sunglasses. We logged the time at which each utterance began and ended, using the auditory signal with the visual signal to assist in identifying the speaking target. For this, we used VideoCoder (1.2), custom software designed for accurately time-stamping events in video. Gaze locations were then categorised according to which target was being fixated and whether they were currently speaking (for full information, see the Supplemental Information and Table S2).

There was an effect of condition (*F*(1, 39) = 139.010, *p* < .001, η^2^ = .781), indicating that participants made more fixations to speaking targets in the sunglasses condition (*M* = 54.1% of fixations on the current speaker, *SD* = 4.1%) than in the control condition (*M* = 46.8%, *SD* = 5.7%). There was no effect of group (*F*(1, 39) = 2.493, *p* = .122, η^2^ = .060) and no interaction between condition and group (*F*(1, 39) = 0.531, *p* = .470, η^2^ = .013).

We then analysed at which point in time participants made a fixation to a speaker. The start times of each utterance (taken from each target in each clip) were used to create 10-ms bins ranging from −1000 ms prior to speech beginning to 1000 ms post-utterance beginning. We then compared these bins to the fixation data and coded bins as to whether they contained a fixation on a target speaking, a fixation elsewhere, or no fixation. We extracted a percentage of looks to a speaker for each bin (averaged across participant, condition and the multiple utterances), which we could then compare within the time period of interest. The result was an estimate of the probability of looking at a speaker, time-locked to the beginning of their speech. We have previously shown that participants sometimes move in advance of the change in speaker, and that the timecourse is affected by both auditory and visual information (Dawson & Foulsham, 2021).

**Figure 2.**
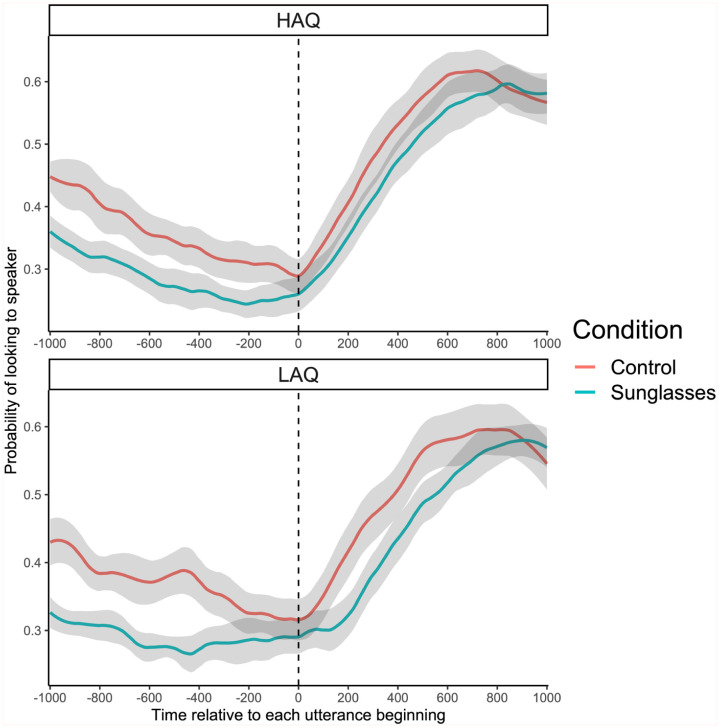
Probability of fixations being on the speaker, relative to when they started speaking. *Note.* Lines plot the mean across participants, smoothed over time, with shading indicating the 95% confidence intervals. A time of zero (dotted vertical line) indicates the time at which a speaker began speaking.

We then analysed the probability of fixations to a target speaker upon the utterance beginning. This analysis was calculated across all data points from the 201 time frames (10 ms bins; −1000 to +1000 ms) from each participant and each condition giving a total of 16,482 data points to analyse. The probability of fixations landing on the target speaker, relative to when they started speaking, can be seen in Figure 2. This provides a test of whether autistic traits, and sunglasses, change the timing of fixations to speakers around the time of a new utterance. The graph shows how the relative frequency of looks to a target increases around the time that they begin talking – a pattern seen in both groups and both conditions.

When examining the overall probability of fixations within this time frame, a mixed ANOVA established that there was an effect of condition (*F*(1, 39) = 97.756, *p* < .001, η^2^ = .715). Thus, around the time of the beginning of speech, there were more looks to a speaker in the control (HAQ: *M* = 0.44, *SD* = 0.04; LAQ: *M* = 0.43, *SD* = 0.04) than in the sunglasses (HAQ: *M* = 0.38, *SD* = 0.03; LAQ: *M* = 0.36, *SD* = 0.02) condition, which confirms our hypothesis that visible eyes are important for smoothly following the speaker. There was no significant effect of group (*F*(1, 39) = 1.954, *p* = .170, η^2^ = .048). The interaction between condition and group was also not significant (*F*(1, 39) = 1.218, *p* = .276, η^2^ = .030), indicating that both groups responded to the two conditions in a similar way.

**Figure 3.**
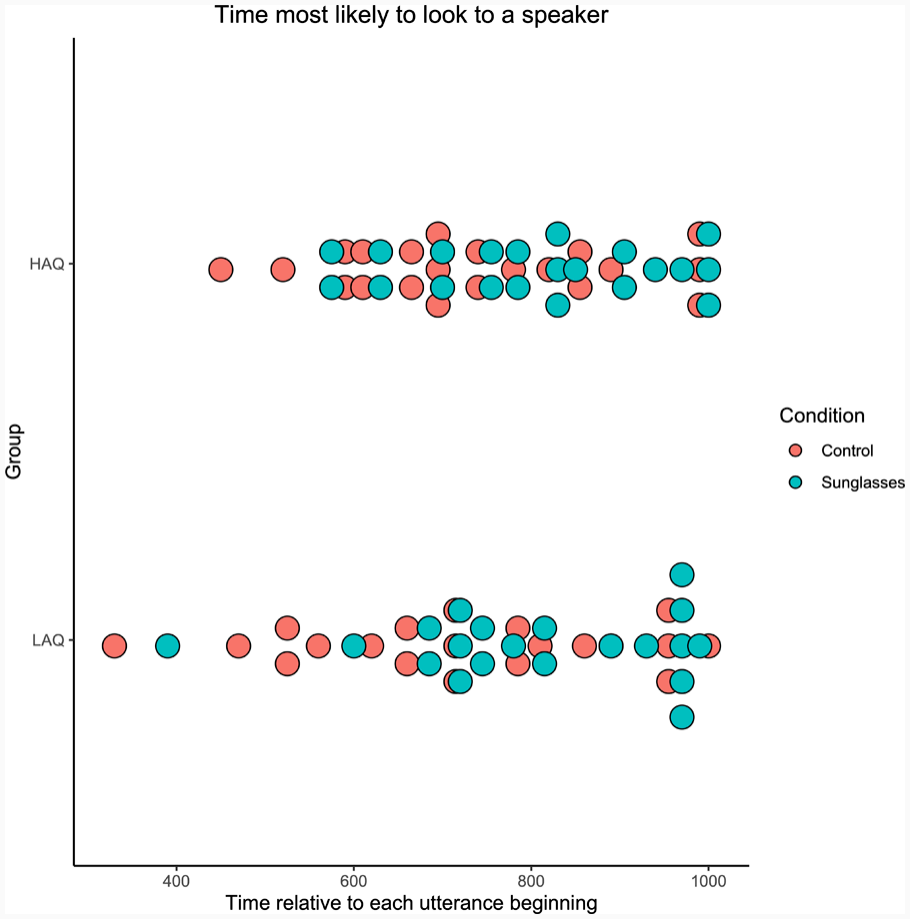
The average time at which participants were most likely to be looking at a speaker, split by condition and group.

In order to analyse how early gaze moved towards the speaker, we calculated at which point in time (−1000 ms to +1000 ms post-utterance beginning) each participant was most likely to be looking at a speaker. We analysed this by finding the time bin with the maximum probability for each participant (see Figure 3).

A mixed ANOVA established that there was no effect of group on peak fixation time (*F*(1, 39) = 0.035, *p* = .852, η^2^ = .009). There was a significant effect of condition (*F(*1, 39) = 5.22, *p* = .028, η^2^ = .118) and a non-significant interaction (*F*(1, 39) = 0.013, *p* = .909, η^2^ < .001). Participants looked at speaking targets earlier, relative to the start of the utterance, in the control condition (HAQ: *M* = 735 ms, *SD* = 155 ms; LAQ: *M* = 724 ms, *SD* = 184 ms) compared to the sunglasses condition (HAQ: *M* = 807 ms, *SD* = 138 ms; LAQ: *M* = 803 ms, *SD* = 156 ms). The AQ group had no effect.

This result may seem to conflict with the percentage of overall looks to speakers, where there was more time spent on the speakers in the sunglasses condition. The results can be reconciled by considering the time window around the speaker beginning their utterance. Although viewers in the sunglasses condition looked at speakers for longer, they were less sensitive to the beginning of speech (the turn-taking between speakers). In the control condition, participants shifted earlier, which may have meant reduced looking at speakers overall because in some cases they looked away before the next turn in the conversation.

## Discussion

In this experiment, we considered how eye fixations were deployed during the watching of conversations. If this reflects complex social attention, then we should expect it to be sensitive to the presence of social cues in the video, and to individual differences in the response to these cues. In particular, we investigated whether those with high levels of autistic traits (HAQ) would view the conversation differently from those with low levels of autistic traits (LAQ), and we used sunglasses to obscure the eyes of the conversants in half of the clips. There were a number of interesting findings.

First, we found a difference between LAQ and HAQ participants, which was specific to the particular regions that were fixated. Both groups spent most of the time fixating the people rather than the background, and they also showed similar patterns in terms of looking at the person who was currently speaking. However, high-trait individuals made fewer fixations on the eyes and mouth than low-trait individuals. Since their overall proportion of fixations on the targets did not differ, this indicates that HAQ participants spent more time on other regions of the face and body, perhaps indicating less sensitivity to socially important cues. Previous research has reported that autistic individuals, and those with high levels of autistic traits, look less at faces and eyes (Chita-Tegmark, 2016; Dalton et al., 2005; Klin et al., 2002). However, this pattern of results has not always been observed, particularly in complex video stimuli (Forby et al., 2023; Hedger et al., 2020). The addition of sunglasses did not interact with the difference between groups. The fact that HAQ individuals looked less at the face, therefore, does not seem to have been ameliorated by sunglasses (as it might if the eye was aversive), and the difference remained even when some of the social gaze information was obscured.

Second, sunglasses made a clear difference to how the videos were attended, and this difference impacted both overall fixations and the timing of gaze to the person speaking. Participants looked more at the eyes of the people in the clips, and at the person who was currently speaking, when that person was wearing sunglasses. This is counterintuitive, since we might expect fixating the eyes to be less useful when fine-grained information on gaze direction and expression is obscured. It may be that participants looked at the sunglasses since they were novel and unexpected, or that they spent more time on speakers with sunglasses because it was more difficult to understand the flow of the conversation in this condition. Indeed, participants appeared to be foraging for information about gaze, by looking at the eye region more in the sunglasses condition even though there were no eyes to find. In this respect, both HAQ and LAQ groups showed the same pattern of increased looks in the sunglasses condition.

Critically, when we zoomed in on the time around the change in speaker, it was actually in control clips, where information from the eyes was available, that participants spent more time fixating the speaking target. When the eyes were visible, participants were quicker to shift gaze to a new speaker in comparison to the sunglasses condition. This is novel evidence that fine-grained information from the eyes is an important signal for smooth turn-taking in conversation.

A possible limitation of Experiment 1 is that, although none of the participants had a clinical diagnosis, autism and autistic traits often overlap with other disorders such as ADHD. In Experiment 2, we replicate the experiment with a group of participants who were pre-screened for ADHD symptoms. Concentrating on a conversation is a realistic activity which individuals with ADHD might find difficult. However, there is little existing research on gaze in this context and individual differences in ADHD. We therefore asked whether complex social attention will manifest differently in groups with different levels of ADHD symptomology.

## Experiment 2

### Method

Experiment 2 was a partial replication of Experiment 1, but this time with participants who fall into high and low trait groups in terms of ADHD symptomology.

#### Participants and ADHD Classification

In a first step, 248 students from University of Essex were asked to complete the Adult ADHD Self-Report Scale (ASRS; Kessler at al., 2005) via an online questionnaire. We used this symptom checklist to classify participants with high (H-ADHD) and low (L-ADHD) levels of ADHD traits. The ASRS consists of 18 items, which participants score on a Likert-type scale from 0 to 4. We summed all the items together to produce an overall measure of strength and frequency of ADHD symptoms. Scores in our sample ranged from 13 to 61, and the mean (*SD*) score was 37.87 (12.50). There is not a clear clinical cut-off for this questionnaire (Kessler et al., 2005). We therefore used the upper and lower quartiles of our pre-screened sample to recruit participants with high or low levels of ADHD symptoms. The lower quartile included scores from 13 to 34 whereas the upper quartile included scores between 45 and 61. We continued recruiting from these subgroups for our eye-tracking study until we reached a sample size of 20 for each. This sample size was pre-registered (see Experiment 1 for sensitivity analysis and further justification).

In the H-ADHD group (10 females; ages 18–29, *M* = 21.15, *SD* = 1.48), 2 participants reported being diagnosed with ADHD. In the L-ADHD group (17 females; ages 18–31, *M* = 22.6, *SD* = 3.87), no participants reported a diagnosis of ADHD. No participants in either group reported taking psychostimulants or being diagnosed with autism. All of the subjects reported normal or corrected-to-normal vision. Participants were awarded five pounds for their participation. Ethical approval was received from the University of Essex, and participants gave their written, informed consent.

#### Apparatus, Stimuli and Procedure

The apparatus, stimuli, and procedure were the same as in Experiment 1. Participants watched the set of pre-recorded conversations, half of which featured target individuals wearing sunglasses.

#### Data Analysis

The same approach was adopted as in Experiment 1. One participant (from the H-ADHD group) was excluded due to several trials in which eye data were missing for more than 50% of the time. No other trials were removed, and non-transformed participant means were submitted to statistical analysis. General viewing behaviour is described in the Supplemental Information and was very similar to that in Experiment 1, with no overall differences between groups and almost all fixations on the targets. Analysis of dwell time is also presented in the Supplemental Information.

### Results

#### Fixations to Targets’ Eyes and Mouth

Table 2 shows the average percentage of fixations to targets’ eyes and mouth and elsewhere on the target throughout the clips.

**Table 2.** The Mean Percentage of Fixations to the Eyes, Mouth and Elsewhere on the Target in Experiment 2, Split by Group (Low and High Levels of ADHD Traits) and Condition (Control and Sunglasses).

Group	Statistic	Mean % fixations to targets					
		Control			Sunglasses		
		Eyes	Mouth	Elsewhere	Eyes	Mouth	Elsewhere
H-ADHD	M	25.45	16.03	58.52	26.21	16.49	57.30
	SD	18.28	15.48	16.15	18.16	14.77	16.69
L-ADHD	M	24.62	21.01	54.38	26.05	19.89	54.06
	SD	17.39	14.39	18.32	16.77	14.12	18.53

*Note.* Fixations outside the main target ROIs are not included here. ROI = Region of interest; ADHD = Attention Deficit Hyperactivity Disorder; H-ADHD = High levels of ADHD traits; L-ADHD = Low levels of ADHD traits.

These values were entered into an ANOVA with within-subject factors of condition (sunglasses or control), ROI (eyes and mouth) and the between-subjects factor of group. Although there were numerically more fixations on the eyes than the mouth, this difference was not significant (*F*(1, 37) = 2.818, *p* = .102, η^2^ = .071). There was also no significant effect of condition (*F*(1, 37) = 0.331, *p* = .569, η^2^ = .009) or group (*F*(1, 37) = 0.460, *p* = .102, η^2^ = .071), and there were no significant interactions (all *F* < 1). This indicates that the eyes and mouth regions were fixated similarly regardless of group and condition. This pattern is different from Experiment 1, where the addition of sunglasses led to more looks to the eyes. It also means that we can reject the hypothesis that high traits of ADHD affect looks to the eyes (in the same way as autistic traits).

#### Fixations to Speakers

We used the same record of utterances as in Experiment 1 to analyse when targets were speaking and whether the observer was looking at the speaker (see Supplemental Information for more details). There was an effect of condition (*F*(1, 37) = 41.378, *p* = .001, η^2^ = .528) with more fixations on the speaker in the sunglasses condition (*M* = 52.4%, *SD* = 6.3%) than in the control clips (*M* = 46.2%, *SD* = 5.3%). There was no effect of group (*F*(1, 37) = 0.337, *p* = .565, η^2^ = .009) and no condition × group interaction (*F*(1, 37) = 0.298, *p* = .588, η^2^ = .008). This replicates the pattern observed in Experiment 1.

**Figure 4.**
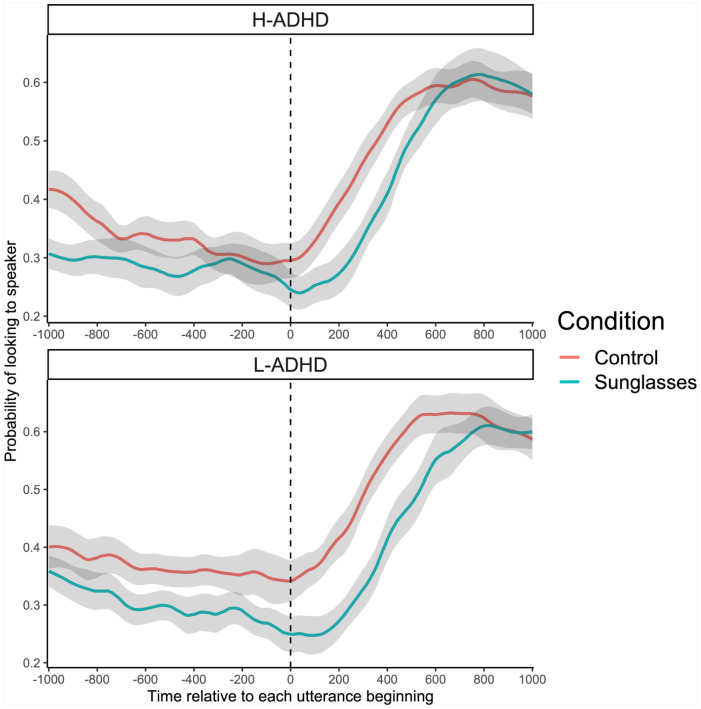
The probability of fixations being on the speaker in Experiment 2, relative to when they started speaking and averaged across condition and group.

As in Experiment 1, we analysed the average probability of fixations to a speaker around the time of each target’s utterance. This analysis across all data points from the 201 time bins, from each participant and each condition, gave a total of 15,678 data points. The timecourse of fixation probability relative to target speaking onset can be seen in Figure 4. This pattern is similar to that from Experiment 1.

A mixed ANOVA on the overall probability of fixations within this time frame established that there were significant differences between ADHD groups (*F*(1, 37) = 5.231, *p* = .028, η^2^ = .124), conditions (*F*(1, 37) = 107.993, *p* < .001, η² = .745) and a significant interaction (*F*(1, 37) = 4.966, *p* = .032, η^2^ = .118). Pairwise comparisons with a Bonferroni correction demonstrated that there were significant differences between conditions for both the L-ADHD group (control: *M* = 0.45, *SD* = 0.03; sunglasses: *M* = 0.38, *SD* = 0.02; *p* < .05) and the H-ADHD group (control: *M* = 0.42, *SD* = 0.03; sunglasses: *M* = 0.37, *SD* = 0.03; *p* < .05). There were significantly more looks within this timeframe in the control than in the sunglasses condition. This was particularly true in the L-ADHD group. Thus, unlike the overall analysis, this timing analysis was sensitive to some small differences in behaviour between groups.

**Figure 5.**
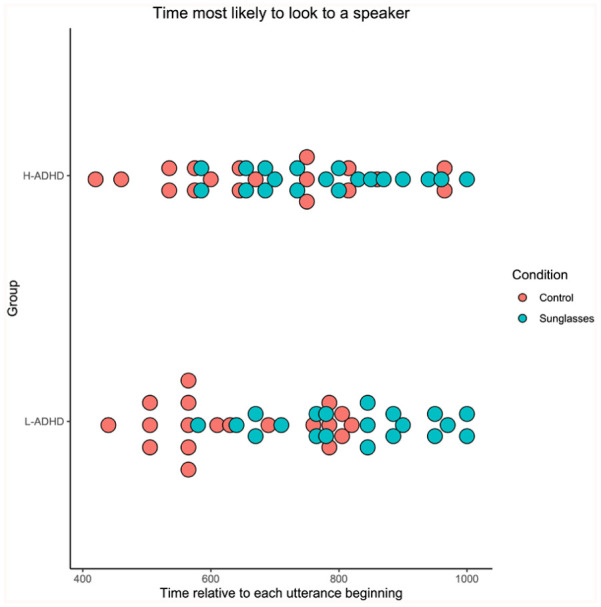
The average time at which participants were most likely to be looking at a speaker in Experiment 2, split by condition and group.

We also quantified the point in time (from −1000 ms to +1000 ms post-utterance beginning) when the participant was most likely to be looking at a speaker (the 10 ms bin with the maximum percentage for each participant; see Figure 5). A mixed ANOVA established that there was a non-significant effect of group (*F*(1, 37) = 0.088, *p* = .768, η^2^ = .002). There was a significant effect of condition, (*F*(1, 37) = 13.82, *p* < .001, η^2^ = .272), and a non-significant interaction (*F*(1, 37) = 1.249, *p* = .271, η^2^ = .033). Participants were more likely to look at a target earlier with respect to when the utterance began in the control condition (L-ADHD: *M* = 662 ms, *SD* = 148 ms; H-ADHD: *M* = 690 ms, *SD* = 157 ms) compared to the sunglasses condition (L-ADHD: *M* = 822 ms, *SD* = 125 ms; H-ADHD: *M* = 776 ms, *SD* = 123 ms). ADHD group had no reliable effect.

Overall, as in Experiment 1, looks were more likely and earlier in the control compared to the sunglasses condition in the timeframe around targets beginning their utterance.

## Discussion

In this experiment, we were interested in whether those with high levels of ADHD traits would view the conversation differently, as well as how viewing was affected by sunglasses. In general, behaviour was highly similar to Experiment 1. Clips where the eyes were occluded with sunglasses led to more fixations on the current speaker, but they also led to fewer, slower fixations on this person around the beginning of their utterance. We did not replicate the increase in looks to the eyes in the sunglasses condition that we observed in Experiment 1. This finding should therefore be viewed with caution.

Unlike the differences we observed in looks to eyes/mouth with autistic traits, there were no differences in behaviour between ADHD high and low symptom groups. Both groups looked more at the eyes than the mouth, and at the speaker about 50% of the time (more than would be expected by chance given that there were three targets). These patterns were very consistent with Experiment 1. It may be that ADHD does not have an effect on behaviour in this context, and indeed prior studies have indicated that ADHD alone is not associated with changes in social attention seen in autism, or when the two are comorbid (Groom et al., 2017; Seernani et al., 2021). Of course our participants were selected with high levels of ADHD symptoms, and not with a clinical diagnosis, and so behaviour in a clinical group might be different.

Interestingly, there was an interaction between occlusion by sunglasses and ADHD group. This came about only when we looked in detail at gaze to the speaker around the time of their utterance. At this time, high trait individuals showed slightly fewer fixations on the speaker and less of a difference between control and sunglasses clips. This might indicate that the high trait group were finding it more difficult to focus on the subtle cues which indicate a change in speaker. Previous research has indicated that participants with clinically diagnosed ADHD are less effective in inhibition and preparation to perform a new task (Cepeda et al., 2000; Luna-Rodriguez, 2018). This might translate into reduced flexibility when changing between speakers or between the two types of clips presented here.

## General Discussion

Research has reported a strong bias for participants to orient to faces and eyes, and follow the gaze of depicted individuals (End & Gamer, 2019; Flechsenhar et al., 2018; Friesen & Kingstone, 1998). A bias towards the eyes has also been observed when using dynamic stimuli (Foulsham et al., 2010; Foulsham & Sanderson, 2013; Freeth et al., 2013; Klin et al., 2002). The present study provided an innovative way to explore this bias, within the context of observation of realistic conversations. The theoretical motivation for this was to examine, through the addition of sunglasses which occluded the eyes, whether fine-grained information on gaze was responsible for people fixating the eye region, and whether removing this information had an impact on how participants followed the depicted interaction. Across both experiments, participants spent almost all of the time looking at the people in the clips, and this general finding was unaffected by the addition of sunglasses. However, detailed analysis of when and where participants looked showed that eye occlusion mattered for how participants followed the conversation. There were also differences according to the level of autism and ADHD traits. This indicates that the paradigm is indeed sensitive to differences in social attention.

Participants looked more at the eyes than at the mouth, in both experiments and in all conditions. This bias was not reduced with sunglasses, and in fact it was accentuated (but only significantly so in Experiment 1). Participants continued to look to the eye area in the sunglasses condition, despite not being able to view precise information about gaze or emotion. Why did participants continue to prioritise the eyes? We suggest that looking at the eyes is a habitual behaviour which normally confers advantages in terms of extracting information (and sending signals in real face-to-face interaction). If participants were foraging for information from the eyes, and this was prolonged when the information was occluded by sunglasses, it would have taken effort to disengage and move elsewhere. Interestingly, this volitional process seems to have been equally present in individuals with high traits of autism and ADHD.

We have previously shown that participants show some of the same gaze behaviour when watching video speakers as they do when actually in a conversation (such as looking at the speaker when listening; Dawson & Foulsham, 2021). It may be that making eye contact with someone wearing sunglasses during a face-to-face interaction is particularly important since their own signals are masked. This could also explain the fact that participants fixated the person currently speaking more often when the actors were wearing sunglasses. It may also have been that participants followed these speaking participants more closely in the sunglasses condition because they had to rely more on gesture and other cues, in the absence of gaze cues. The increase in looking to the eyes of targets wearing sunglasses was only found in Experiment 1. Although the participants involved were similar in both experiments, we cannot rule out the possibility that the difference in samples might impact this specific result. Participants in Experiment 1 were Canadians tested in Canada, watching British targets. In contrast, in Experiment 2, both participants and the targets in the clips were British. It is therefore possible that a cultural difference made the sunglasses particularly stand out in Experiment 1.

We found changes in the shifting between speakers with the addition of sunglasses, and we replicated these results in Experiment 2. In both experiments, participants watching sunglasses clips moved their eyes less often to the speaker at the time at which there was a change in speaking turn. They were also slower to do this than when the eyes were visible. If we assume that looking at the speaker is a useful thing to do, then sunglasses led to a less smooth transition to a new speaker. Since sunglasses led to more fixations on speakers overall, it seems that removing the information from the eyes had a specific effect on the transition from one speaker to the next, perhaps by leading to uncertainty about who will speak next and when. In face-to-face interactions between two people, there are clear gaze signals which indicate the change in speaker and listener (Ho et al., 2015), and there is some evidence that these are also seen when a participant responds to prerecorded speakers (Freeth et al., 2013). It has also been reported that participants in a real interaction use the gaze cues of others (e.g. when interacting in a shared task; Macdonald & Tatler, 2018). The present results provide unique evidence that fine-grained information from the eyes is used in multi-party conversation to follow the speaker.

A possible limitation of our design is that the clips in the two conditions (sunglasses and control) were not identical. Although both sets of clips were recorded with the same people and situation, the exact conversations and body language could have been different, which could have influenced the results. For this reason, future research could manipulate videos post hoc (e.g. digitally altering to mask the eyes) or use artificially generated videos or avatars. While the evidence above suggests that some of the same patterns regarding fixations between speakers occur both on video and in real life, there remain many differences between the two (see López et al., 2023), and sunglasses and eyecoverings could provide a realistic way of studying gaze cues in face-to-face settings.

We also investigated potential differences in social attention in those with high and low traits of two common neurodevelopmental conditions (autism and ADHD). The effects of autism and related traits on eye-tracking measures of social attention have not always been consistent (Forby et al., 2023; Hedger et al., 2020), but here we found, even in a non-diagnosed sample, that those with higher traits of autism were less likely to look at the eyes and mouth. In López et al. (2023), the experimenters measured fixations to videos of people sitting in silence. Both autistic and non-autistic groups took part, and in one condition, the participant believed that they were watching a live webcam feed rather than a pre-recorded video. The results showed that non-autistic viewers spent more time fixating the face than autistic viewers, but only in the video condition. When both groups believed that they were watching a live feed, the non-autistic observers looked less at the people, and in that condition, there was no difference between groups. In the present study, participants knew that the video was pre-recorded. Thus, while the difference we report partly replicates that in López et al., that study also underlines that beliefs and norms around the source of the video are important. In our case, participants with low levels of autistic traits looked more at the eyes, and this could be because they were not concerned by the same social norms that would be important in real interactions. It is not yet known how specific patterns of eye and mouth looking might differ in real face-to-face settings.

Those with high and low levels of ADHD symptoms mostly viewed clips in the same way, which is not what we would expect if such symptoms reflect general difficulties in concentration and inattention. We also found few effects of individual differences in ADHD symptoms in a recent study of social attention under cognitive load (Martinez-Cedillo et al., 2022). Although there is a previous report of children with ADHD spending less time fixating the eyes and mouth of images (Serrano et al., 2018), we found no difference between ADHD trait groups with our dynamic stimuli (for either the proportion of fixations, or the proportion of time; see Supplemental Information). We did however find a subtle difference in the timecourse of fixations to speakers, which suggested that those with high ADHD traits shifted to the new speaker less often. This should be examined in a larger or diagnosed sample.

Despite some notable differences between the high- and low- trait groups, in both experiments, we must remain cautious in equating these participants with diagnosed individuals, who may show heterogeneous behaviour, particularly in the case of ADHD where multiple subtypes have been identified. Our limited sample size in each experiment means that smaller effects would have been unlikely to be detected (although selecting high- and low-trait groups from a larger pre-screened pool should have led to a more powerful design). Another limitation is that we did not control for the sex of participants, who were largely female. This reflects the university population that we sampled from, but may be atypical for autism (see Harrop et al., 2018, for discussion of sex differences and attention in autism). The effects of sunglasses were replicated in Experiment 2, and thus were observed across two samples in different countries (Canada and the UK). Nevertheless, all of our participants were students from Western universities, which provides constraints on the generality of our findings.

Finally, we must also be cautious with regards to the independence of autism, ADHD and their associated traits. As discussed, diagnoses of autism and ADHD frequently co-occur. Correlations between autistic trait measurements and ADHD symptoms in the general population have been published and range from around 0.1 to 0.4 (Lundin et al., 2019; Sun et al., 2024). It is therefore possible that the HAQ group in Experiment 1 also had high traits of ADHD (and vice versa in Experiment 2). This would mean that differences between high- and low-trait groups, particularly the effect of autistic traits in Experiment 1, might be contaminated by the influence of ADHD. Unfortunately, concurrent measures of the two traits were not taken for the current study. It seems unlikely that the effect of autistic traits on looks to the face in Experiment 1 is due to ADHD traits for at least two reasons. First, we did not find the same pattern of results in Experiment 2, where the influence of ADHD traits should have been much stronger since participants were selected using the ASRS. Second, the pattern of reduced looks to the face and eyes has previously been reported in many other studies with both autistic individuals and those with high traits (Chita-Tegmark, 2016). Nonetheless, we recommend that future studies pay more heed to the overlap between these two sets of traits.

## Conclusions

Our experiments were designed to test the effect of occluding the eyes in conversation following while comparing two population groups of interest. Occluding the targets’ eyes with sunglasses affected the timecourse of looks, with participants slower to fixate a speaker upon the utterance beginning. We suggest that this was due to the inability to follow the targets’ signalling cues (their eyes), which impeded conversation following. Those high in autistic traits were less likely to look at the key features of the face, but this was not specific to non-occluded eyes. Following a conversation is a difficult challenge for human visual attention, and differences in the timing of this behaviour can reveal insights about the cues involved and about clinically-relevant individual differences. This paradigm, which involves manipulating eye occlusion of pre-recorded conversations, is sensitive to differences in social attention between different groups of individuals, and promises to be an effective research tool for future investigations of human cognition and attention in dynamic real-world social interactions.

## Supplemental Material

sj-docx-1-qjp-10.1177_17470218251390498 – Supplemental material for Social Attention Through a New Lens: Autistic and ADHD Traits and Eye Occlusion Affect Gaze During Conversation Watching Supplemental material, sj-docx-1-qjp-10.1177_17470218251390498 for Social Attention Through a New Lens: Autistic and ADHD Traits and Eye Occlusion Affect Gaze During Conversation Watching by Jessica Dawson, Astrid P. Martinez-Cedillo, Leilani Forby, Bradley Karstadt, Alan Kingstone and Tom Foulsham in Quarterly Journal of Experimental Psychology
