# Supplementary material for: Social Attention Through a New Lens: Autistic and ADHD Traits and Eye Occlusion Affect Gaze During Conversation Watching
Source: Q J Exp Psychol (Hove). 2025 Oct 16;79(5):1206–21. doi: 10.1177/17470218251390498 (PMC13061312; doi:10.1177/17470218251390498)
Supplement: sj-docx-1-qjp-10.1177_17470218251390498 – Supplemental material for Social Attention Through a New Lens: Autistic and ADHD Traits and Eye Occlusion Affect Gaze During Conversation Watching [file sj-docx-1-qjp-10.1177_17470218251390498.docx]

**Supplementary Information**

**Social attention through a new lens: Autistic and ADHD traits and eye occlusion affect gaze during conversation watching**

Jessica Dawson^1^, Astrid P. Martinez-Cedillo^1,2^, Leilani Forby^3^, Bradley Karstadt^3^, Alan Kingstone^3^, & Tom Foulsham^1*^

^1^Department of Psychology, University of Essex, Colchester, United Kingdom.

^2^Department of Psychology, University of York, United Kingdom

^3^Department of Psychology, University of British Columbia, Vancouver, British Columbia, Canada

These additional analyses can be read, in combination with the main paper, as an additional description of behaviour in the conversation-watching task.

**Experiment 1**

*General viewing behaviour*

We examined how participants responded to the conversation clips by analysing any global differences in the way the two groups moved their eyes. The HAQ group made slightly fewer fixations per clip (*M* = 76.82, *SD* = 12.51) than the LAQ group (*M* = 84.43, *SD* = 16.31). These fixations were also shorter on average (Mean fixation duration = 388.43 ms, *SD* = 83.19 and 361.10 ms, *SD* = 97.35 for HAQ and LAQ, respectively). However, independent sample *t*-tests indicated that these differences were not significant for either number of fixations, (*t* (39) = 1.689, *p* =.10, *d* = 0.524), or average fixation duration (*t* (39) = -0.96, *p* =.33, *d* = 0.15).

*Fixations to targets*

In the main analysis we examined how each group fixated the targets in the scene. We defined a region of interest (ROI) around each target individual. Pooling ROIs together, participants in both groups and conditions spent almost all of their fixations on the people in the scene (an average of 98%).

The average percentage of fixations on the ROIs was entered into an analysis of variance (ANOVA) with the within-subjects factor of condition (Sunglasses or Control) and the between-subjects factor of group (HAQ or LAQ). There was no effect of condition (*F* (1, 39) = 2.192, *p* = .147, η² = .053), or group (*F* (1, 39) = 0.070, *p* = .793, η² = .002), and no interaction between condition and group (*F* (1, 39) =0.638, *p* = .429, η² = .016). Thus both high and low trait individuals looked mostly at the people in the clips rather than the background.

*Dwell time on facial features*

Table S1 shows the proportion of time that participants spent on the eyes and mouth in Experiment 1. These values correspond to the total duration of all fixations on the region, divided by the total trial time. Repeated measures ANOVA showed a similar pattern as found with the proportion of fixations (as reported in the manuscript). There was a significant effect of group (*F* (1, 39) = 6.77, *p* = .013, η² = .148), with the LAQ group dwelling longer on the face than the HAQ group. There was a significant effect of area (*F* (1, 39) = 12.68, *p* < .001, η² = .245) and a marginal effect of condition (*F* (1, 39) = 3.786, *p* = .059, η² = .088). As with the measure of fixation proportion, the bias to look at the eyes vs. the mouth was more pronounced in the sunglasses condition, leading to a marginal area x condition interaction (*F* (1, 39) = 3.02, *p* = .09, η² = .072). All other interactions were non-significant.

|  |  | Mean % Dwell time | | | |
| --- | --- | --- | --- | --- | --- |
|  |  | Control Condition | | Sunglasses Condition | |
|  |  | Eyes | Mouth | Eyes | Mouth |
| HAQ | M | 37.05 | 21.95 | 41.13 | 17.16 |
|  | *SD* | *25.02* | *21.54* | *21.46* | *14.29* |
| LAQ | M | 47.62 | 26.19 | 45.82 | 23.38 |
|  | *SD* | *24.33* | *21.69* | *20.43* | *15.70* |
| **Table S1.** The mean percentage of dwell time to targets’ eyes and mouth, split by group (low and high traits of autism) and condition. | | | | | |

*Fixations to speakers*

Table S2 shows the average percentage of fixations on the person currently speaking.

|  |  | Mean % Fixations | | | |
| --- | --- | --- | --- | --- | --- |
|  |  | Control Condition | | Sunglasses Condition | |
|  |  | Speakers | Elsewhere | Speakers | Elsewhere |
| HAQ | M | 44.38 | 55.62 | 53.19 | 46.81 |
|  | *SD* | *6.61* | *6.61* | *4.27* | *4.27* |
| LAQ | M | 47.13 | 52.87 | 54.91 | 45.09 |
|  | SD | *4.87* | *4.87* | *3.98* | *3.98* |
| **Table S2.** The average percentage of fixations on the current speaker, split by condition and group. The Elsewhere category includes fixations on the other non-speaking targets and any non-target fixations. | | | | | |

**Experiment 2**

*General viewing behaviour*

First, we examined how participants in the H-ADHD and L-ADHD groups responded to the conversation clips by analysing general eye movement behaviour. There was no difference in the mean number of fixations per clip, (H-ADHD: *M* = 78.2, *SD* = 13.3; L-ADHD: *M* = 78.5, *SD* = 11.2; *t* (37) = -0.09, *p* = .92, *d* = 0.015). There was also no difference in the mean fixation duration (H-ADHD: *M* = 390 ms, *SD* = 65 ms; L-ADHD: *M* = 398 ms, *SD*= 57 ms; *t* (37) = -0.37, *p* = .70, *d* = 0.061). This indicates that participants’ general viewing behaviour was similar. The statistics here are also very similar to behaviour in Experiment 1.

*Fixations to targets*

As in Experiment 1, to explore how the participant group and clip condition affected how much participants looked at the targets, we defined a region of interest (ROI) around each target individual. These ROIs accounted for almost all of the fixations in the clip. There was no effect of condition (*F* (1, 37) = 0.107, *p* = .746, η² = .003), or group (*F* (1, 37) = 2.290, *p* = .139, η² = .058), and no interaction (*F* (1, 37) =1.722, *p* = .197, η² = .044). These results indicate that both groups spent most of the time looking at the people and that this did not vary according to sunglasses.

*Dwell time on facial features*

Table S3 shows the proportion of time that participants spent on the eyes and mouth in Experiment 2. There was no effect of condition (*F* (1, 37) = 0.329, *p* = .570, η² = .009), or group (*F* (1, 37) = 0.194, *p* = .662, η² = .005). More time was spent on the eyes than the mouth, but this difference was not statistically reliable (*F* (1, 37) = 1.52, *p* = .225, η² = .040). There were no significant interactions (all *F* < 1).

|  |  | Mean % Dwell time | | | |
| --- | --- | --- | --- | --- | --- |
|  |  | Control Condition | | Sunglasses Condition | |
|  |  | Eyes | Mouth | Eyes | Mouth |
| H-ADHD | M | 30.47 | 19.71 | 30.50 | 21.15 |
|  | *SD* | *22.50* | *18.28* | *23.26* | *19.32* |
| L-ADHD | M | 28.27 | 25.14 | 28.68 | 25.17 |
|  | *SD* | *19.80* | *17.74* | *19.15* | *19.53* |
| **Table S3.** The mean percentage of dwell time to targets’ eyes and mouth, split by group and condition. | | | | | |

*Fixations to speakers*

Table S4 shows the average percentage of fixations on the person currently speaking in Experiment 2.

|  | Mean % Fixations to Speaking Targets | | | | |
| --- | --- | --- | --- | --- | --- |
|  |  | Control | | Sunglasses | |
|  |  | Speakers | Elsewhere | Speakers | Elsewhere |
| H-ADHD | M | 45.59 | 54.41 | 51.65 | 45.11 |
|  | SD | 5.85 | 5.85 | 6.56 | 6.35 |
| L-ADHD | M | 46.77 | 53.23 | 53.24 | 49.57 |
|  | SD | 4.70 | 4.70 | 5.97 | 5.11 |
| **Table S4.** The average percentage of fixations on the current speaker in Experiment 2, split by Condition and Group. The elsewhere category includes fixations on the other non-speaking targets and any non-target fixations. | | | | | |
